# Intrusion of Dacron suture knot 15 years after scleral buckling

**DOI:** 10.1186/s12886-018-0981-1

**Published:** 2018-12-17

**Authors:** Ruiping Gu, Xiujuan Chen, Fang Song, Chunhui Jiang, Haohao Zhu, Gezhi Xu

**Affiliations:** 1 Department of Ophthalmology and Vision Science, Eye and ENT Hospital of Fudan University, 83 Fenyang Rd, Shanghai, 200031 People’s Republic of China; 2 Department of Ophthalmology, Taizhou people’s Hospital, Taizhou, Jiangsu China; 3 Department of Ophthalmology, People’s Hospital of Shanghai, No. 5, Shanghai, 200240 People’s Republic of China; 4 Shanghai Key Laboratory of Visual Impairment and Restoration, Fudan University, Shanghai, 200031 China; 5 NHC Key Laboratory of Myopia (Fudan University), Laboratory of Myopia, Chinese Academy of Medical Sciences, Shanghai, China

**Keywords:** Scleral erosion, Dacron, Scleral buckling surgery, Secondary glaucoma

## Abstract

**Background:**

We present a case of intrusion of a suture knot 15 years after scleral buckling surgery.

**Case presentation:**

A 62-year-old woman with high myopia had undergone scleral buckling surgery in her left eye 15 years previously for rhegmatogenous retinal detachment. She recently displayed highly elevated intraocular pressure, with hyphema and vitreous hemorrhage. After the blood was cleared, a ring-shaped protrusion was noted around the equator of the eyeball, with a blue suture knot standing out on its surface and extending into the vitreous cavity at 5 o’clock. The suture knot was removed successfully. Mass spectrometry revealed that the material of the suture was polyethylene terephthalate, or Dacron. One week later, at the place where the suture knot had been located, the choroidal and retinal tissue disappeared and the silicone buckle remained an uncovered intrusion, whereas the rest of the retina was still attached.

**Conclusions:**

The suture knot was possibly the one used to close the drainage port for subretinal fluid, which was covered by the encircling band. During the buckling procedure, covering a nonabsorbable suture, which is usually placed where the sclera is compromised by trauma or the surgical incision, with an encircling band may lead to the intrusion of the suture. Therefore, a soft absorbable suture may be preferable, if possible.

## Background

‘Intrusion’ is the erosion and protrusion of a scleral suture or implant into the vitreous cavity [1]. Pathological myopia, glaucoma, and scleral buckle tension are strong risk factors for intrusion [2]. Silicone rubber, silicone buckles, silicone sponge, the Arruga suture, and polyethylene tubes have been reported to induce ocular intrusion [1, 3]. Many cases of encircling suture intrusion have been reported in the literature [4, 5]. However, there have been relatively few reports of the intrusion of the suture knot or anchoring suture [6, 7]. Here, we report a case of the intrusion of a Dacron suture knot 15 years after scleral buckling surgery.

## Case presentation

**Fig. 1:**
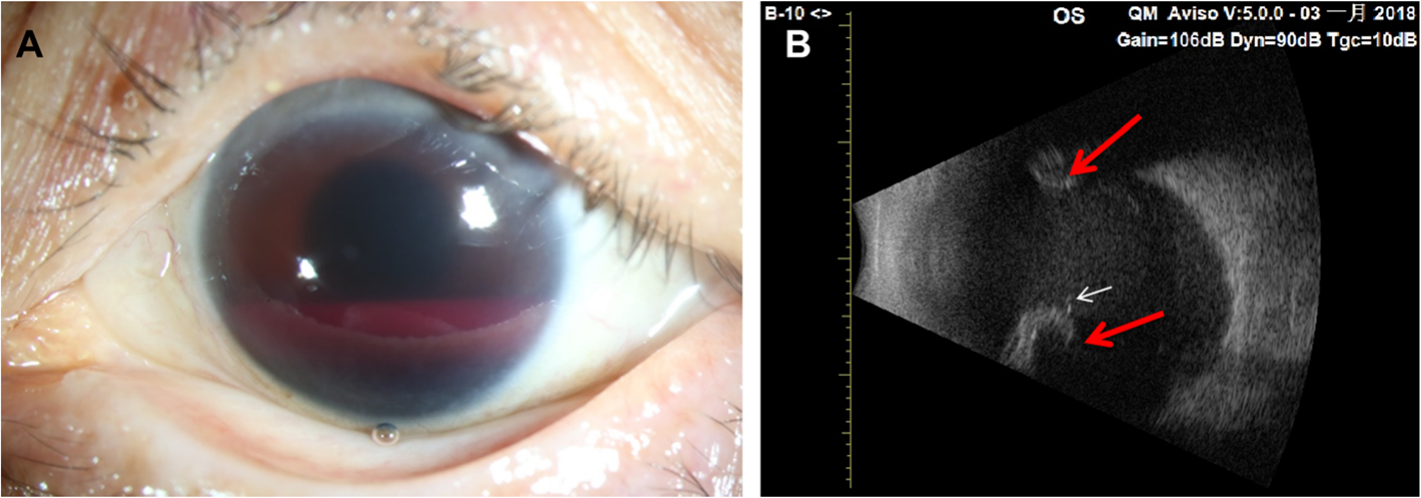
A Anterior segment photograph of the left eye showing diffuse corneal edema and hyphema. B: B-mode ultrasound scan of the patient’s left eye. High-luminance image of the scleral buckle protrusion showing acoustic shadow. Red arrow: protrusion of the scleral buckle; white arrow: acoustic shadow that may represent erosion of the suture node

A 62-year-old woman had undergone vitrectomy 30 years previously for traumatic vitreous hemorrhage, scleral buckling surgery 15 years previously for rhegmatogenous retinal detachment, and phacoemulsification without intraocular lens implantation 1 year previously for cataract in her left eye. She suffered acute loss of vision in her left eye 4 months before admission to our hospital, with pain, left-sided headache, and nausea, and consulted another hospital. Records show that the visual acuity in her left eye was light perception and the intraocular pressure (IOP) was 50 mmHg, and a slit-lamp examination showed diffuse corneal edema associated with hyphema and vitreous hemorrhage. IOP was 25 mmHg after the administration of glaucoma medications (Azopt® Eye Drops containing brinzolamide; Timolol Eye Drops containing timolol maleate; Alphagan® containing brimonidine tartrate), accompanied by the relief of both headache and nausea. The patient had experienced a relapse, with pain, left-sided headache, and nausea, 4 days before attending our hospital. Upon examination, her left eye showed visual acuity of light perception, diffuse corneal edema, hyphema, and vitreous hemorrhage (Fig. 1a), and an IOP of 45 mmHg. B-ultrasound showed vitreous opacities, and a high-luminance indentation of the eye wall with an acoustic shadow could be seen in the vitreous cavity on the B-scan (Fig. 1b). The axial length was 33.70 mm (IOLMaster 500, Zeiss). The patient was diagnosed with secondary glaucoma and treated with lavaging of the anterior chamber and vitreous cavity.

**Fig. 2:**
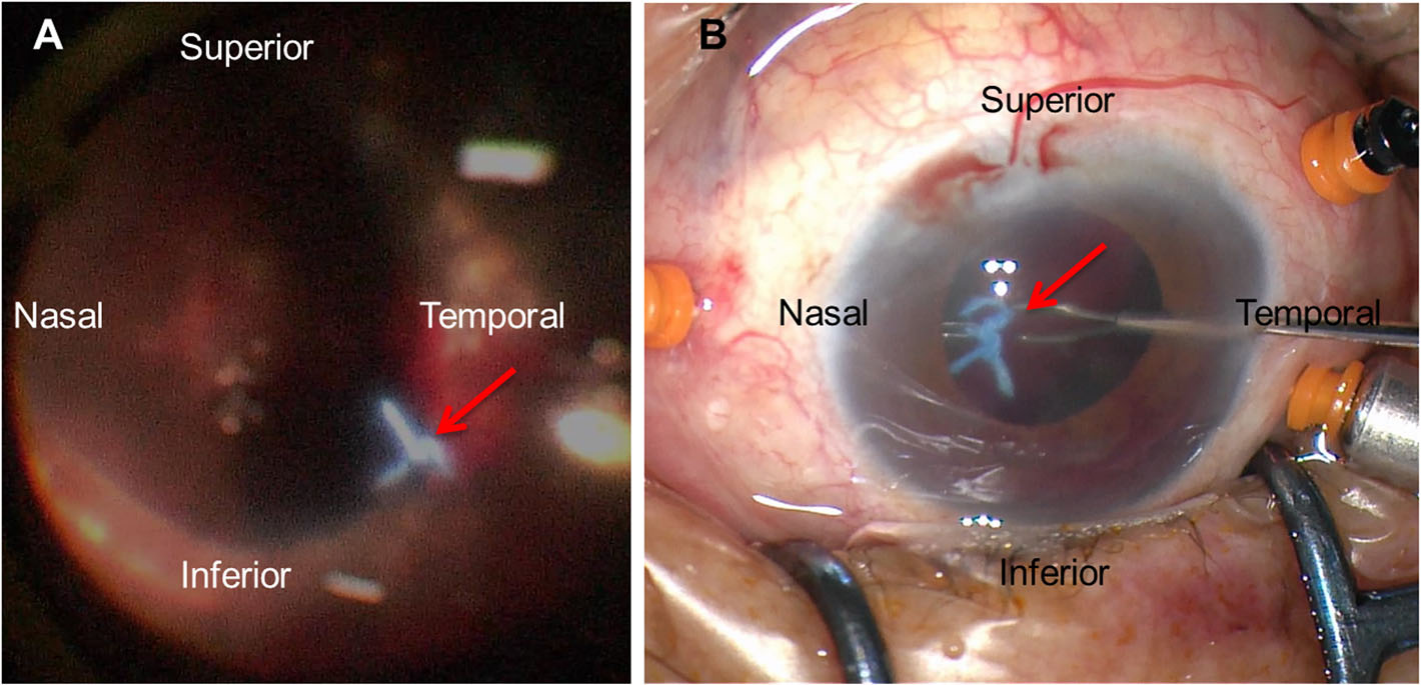
Intraoperative images showing intrusion of the scleral suture node in the left eye. Red arrow: suture node

**Fig. 3:**
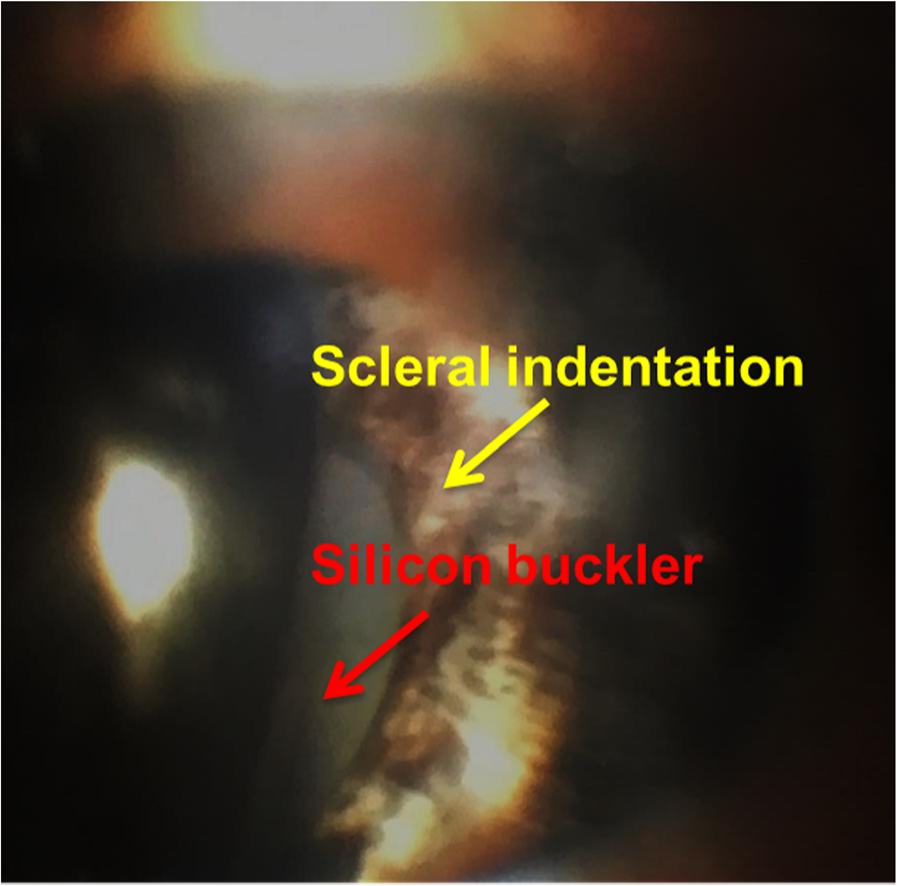
Fundus photograph of the left eye during a slit-lamp examination showing the penetration of the silicon buckle on the inferior temporal segment 1 week after vitrectomy in the left eye. Red arrow: protrusion of the silicon buckle; yellow arrow: scleral indentation

After the vitreous haemorrhage was cleared, a blue suture knot was detected standing out on retinal surface and projecting into the vitreous cavity at 5 o’clock (Fig. 2a). Using forceps, we found that the knot was loosely connected to the underlying tissue, and we removed it with forceps like any other intraocular foreign body, causing no haemorrhage or other complications (Fig. 2b). The next day, the visual acuity in the patient’s left eye was hand motion, the IOP was 14.9 mmHg, and her pain was relieved. One week later, her best correct visual acuity was hand motion and her IOP was 13.6 mmHg in the left eye without anti-glaucoma medication. The vitreous was clear, the retina was still attached, and the ring-shaped protrusion was still visible around the equator. Around 5 o’clock, where the suture knot had been located, the choroid and retinal tissue were missing and the silicone buckle had penetrated into the vitreous cavity (Fig.3). We opted to treat the patient with steroid eye drops and conservative follow-up (observation), unless retinal redetachment or vitreous haemorrhage occurred.

**Fig. 4:**
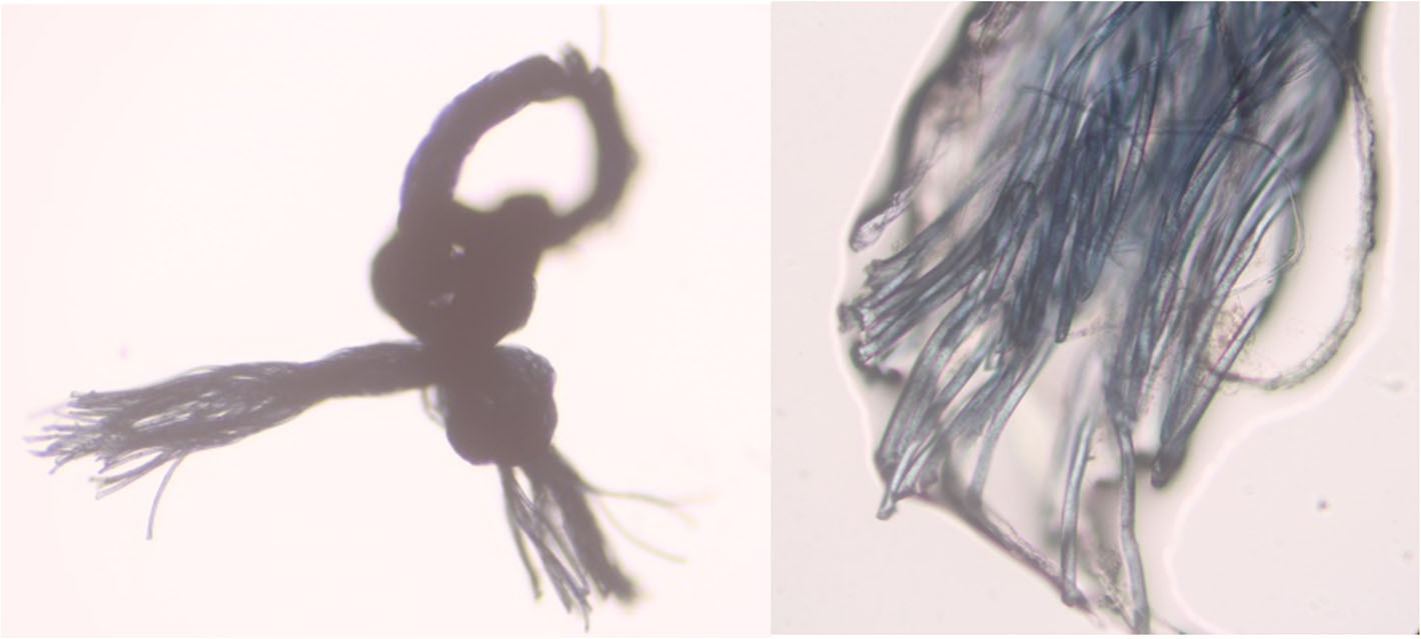
Microscopic view of the suture node

**Fig. 5:**
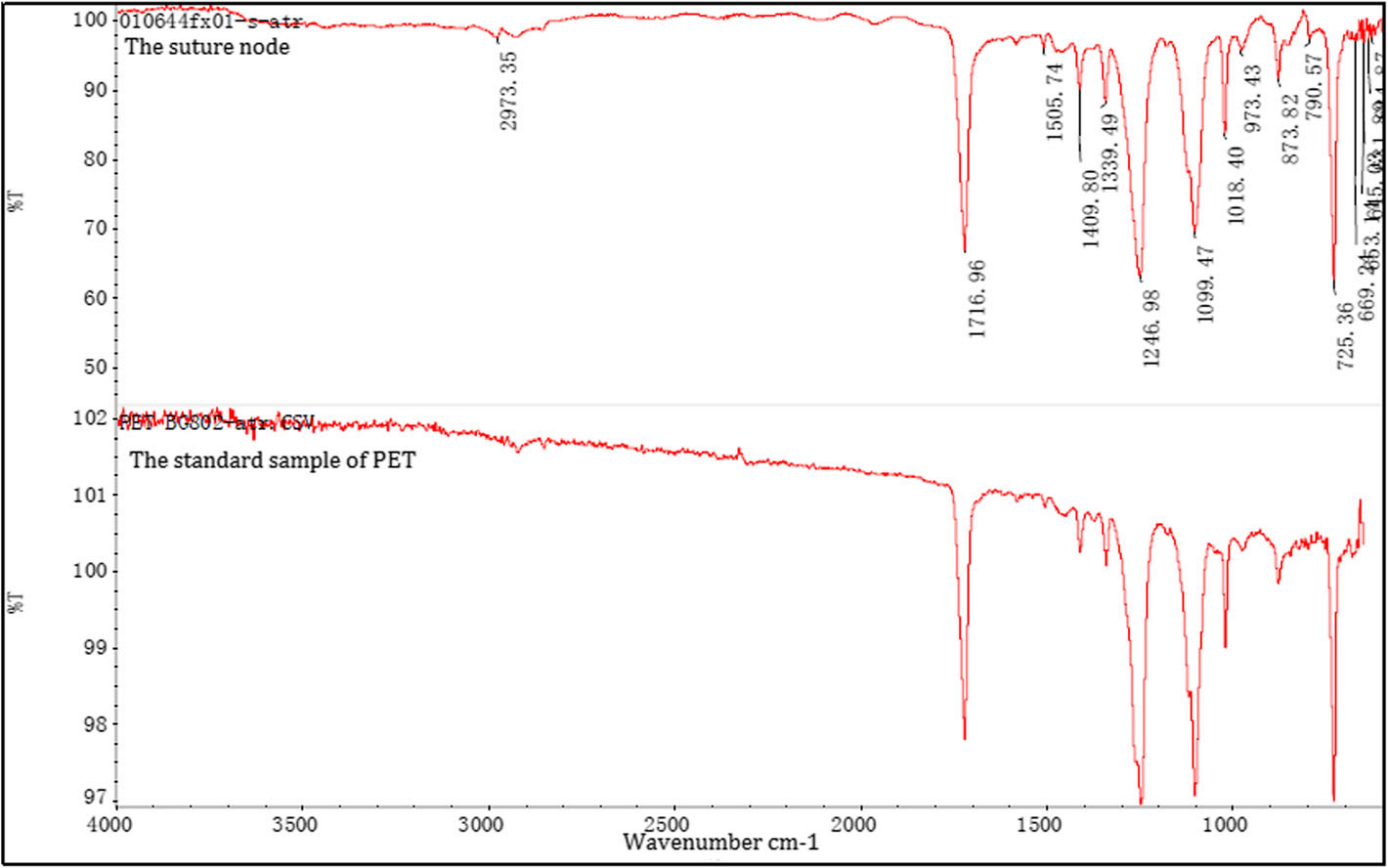
Mass-spectrometric analysis revealed that the material was polyethylene terephthalate (PET)

Microscopy showed the suture as a multifilament of four fibers, each having 11 microfibers of diameter 16 μm (Fig. 4). Mass spectrometry revealed that the material was polyethylene terephthalate (PET) (Fig. 5), possibly Dacron.

## Discussion

Most reports of suture intrusion have concerned encircling sutures [4, 5], which are actually encircling bands. The intrusion of an anchoring suture or suture knot is relatively rare [6, 7]. Here, we have reported a case of the intrusion of a PET suture knot 15 years after scleral buckling surgery.

The manifestations of intrusion vary, from asymptomatic to severe visual loss attributable to vitreous haemorrhage or retinal detachment [1, 2, 8–11]. In our case, the patient presented with highly elevated IOP and eye pain, headache, and nausea, and a clinical examination detected hyphema and vitreous haemorrhage. The intrusion of the suture knot was only found during surgery, so in patients with a history of scleral buckling, the possibility of intrusion must be considered.

An analysis showed that the suture was PET, possibly Dacron, a nonabsorbable suture from Alcon. PET is a compound synthesized from ethylene glycol and terephthalic acid in a polycondensation reaction, and is highly resistant to disintegration when buried in tissue [12, 13]. In our patient, the suture knot was resting on a ring-shaped protrusion, and both the knot and the encircling silicone band were intact. Therefore, it is unlikely that the node was the anchoring suture for the encircling silicone band, but rather that the suture had been used to close the drainage port for subretinal fluid, and was covered by the encircling band. Weinberger reported that the intrusion of anchoring sutures occurs as a late complication of retinal detachment surgery when the extra-scleral buckling technique is used [7]. But in our case, the suture was not the anchoring one. Schepens reported that erosion commences and progresses as the implant is held firmly against the sclera by the sutures, the encircling element, or the solid scar tissue that has grown over the external surface of the implant [1].In our case, the Dacron knot may have been held against the scleral wall by the encircling band. The patient was highly myopic, another risk factor for intrusion. Moreover, the sclera was further weakened by the incision made for the drainage of the subretinal fluid. Cooper et al. reported a case in which a 5–0 Dacron scleral suture knot had eroded into the vitreous cavity 12 years after scleral laceration repair, pars plana vitrectomy, the removal of an intraocular foreign body, and a scleral buckling procedure for penetrating injury [6]. In their case, the Dacron suture was used to close the scleral laceration, and had been covered with a 5 mm radial scleral sponge and overlain with an encircling 240 band. In both Cooper’s and our patients, the encircling band held the suture firmly against a sclera that had been compromised by a traumatic or surgical incision. Therefore, covering a nonabsorbable suture with an encircling band should be avoided in the buckling procedure, especially when the sclera is impaired by trauma, pathological myopia, or surgical incision. If a suture is unavoidable, then a soft, absorbable suture may be preferable.

In conclusion, when covered with an encircling band, a nonabsorbable suture, which is usually placed where the sclera has already been compromised, could erode the sclera and intrude into the vitreous cavity.

## Abbreviations

IOP: intra ocular pressure
PET: polyethylene terephthalate
